# Thermophysical properties and solidification behavior of liquid Vit106a in microgravity

**DOI:** 10.1038/s41526-026-00572-6

**Published:** 2026-02-17

**Authors:** Damien Terebenec, Markus Mohr, Rainer Wunderlich, Hans-Jörg Fecht, Stephan Schneider, Alex Dommann, Antonia Neels

**Affiliations:** 1 Center for X-ray Analytics, Empa Swiss Federal Laboratories for Materials Science and Technology, Dübendorf, Switzerland; 2 Institute of Quantum Technologies, German Aerospace Center (DLR), Ulm, Germany; 3 Institute of Functional Nanosystems, University of Ulm, Ulm, Germany; 4 Institute of Micro and Nanomaterials, University of Ulm, Ulm, Germany; 5 Institute for Frontier Materials on Earth and In Space, German Aerospace Center (DLR), Köln, Germany; 6 ARTORG Center for Biomedical Engineering Research, University of Bern, Bern, Switzerland; 7 Department of Chemistry, University of Fribourg, Fribourg, Switzerland

**Keywords:** Glasses, Engineering

## Abstract

Understanding thermophysical properties such as surface tension (*σ*), total hemispherical emissivity (*ε*), specific heat capacity (*c*_*p*_) and viscosity (*η*) as a function of temperature is essential for optimizing the vitrification of bulk metallic glasses (BMGs). In this study, the thermophysical properties of liquid Vit106a were measured aboard the International Space Station (ISS) using the electromagnetic levitator (EML). The surface tension *σ* exhibited a similar value with other Zr-based BMG, with a weak temperature dependence described by *σ(T)* = 1.557–4.36 ×10^−5^ × (T - 1106) N.m^−1^. The viscosity temperature-dependence *η(T)* was analyzed using the Vogel–Fulcher–Tammann (VFT) equation, yielding a kinetic fragility parameter of D* = 9.8 at high temperature, compared to D* = 21.6 at low temperature, that indicates a fragile-to-strong transition characteristic of Zr-based metallic glass formers. XRD analysis confirms full crystallization of the sample, despite being cooled at a rate of 16 K.s⁻¹, over nine times faster than the critical cooling rate of 1.75 K.s⁻¹ reported in the literature. The crystallized sample reveals a heterogeneous distribution of binary intermetallic phases, including ZrAl_3_, Zr_2_Cu, Zr_2_Ni, ZrAl and Nb_2_Ni. These findings provide insights into the thermophysical behavior of liquid Vit106a for large-scale manufacturing but also raise important questions regarding its good glass-forming ability for larger casting thickness.

## Introduction

Bulk metallic glasses (BMGs) are amorphous metallic alloys that possess exceptional mechanical properties compared to their crystalline counterpart, making them highly attractive for advanced engineering applications. Their peculiar amorphous structure, lacking crystalline defects such as grain boundaries, provides them with high mechanical strength, remarkable elastic strain limits, and excellent corrosion resistance^1,2^. BMGs components are produced by melting the alloy for a long period above its liquidus temperature to ensure complete homogenization. The molten alloy is then rapidly quenched at a critical cooling rate sufficient to suppress crystallization, thereby freezing the atomic structure out of equilibrium in a disordered (amorphous) state. The glass-forming ability (GFA) of a metallic alloy, which is mainly defined by its critical cooling rate, is strongly influenced by its atomic composition. Zr-based BMGs have already shown excellent potential for forming amorphous structures for large casting thickness, such as alloys containing beryllium (Be) with the composition Zr_41_Ti_14_Cu_12.5_Ni_10_Be_22.5_, called Vitreloy1 or Vit1^3^.

To avoid the use of the toxic element Be, several Be-free alternatives with high glass-forming ability have been developed within the Zr-Cu-Ni-Al-*X* system^4,5^, such as the Vit105 (*X* = Ti) alloy, which was the first BMG that has been successfully vitrified within the International Space Station (ISS)^6^. Another good glass-former within the Zr-Cu-Ni-Al-*X* system, also successfully vitrified aboard the ISS, is Vit106 with the composition Zr_57_Cu_15.4_Ni_12.6_Al_10_Nb_5_, where *X* = Nb^7^. Remarkably, just 6 years after its development, the BMG was already integrated into NASA’s Genesis mission as a solar wind collector^8^. Vit106a (Zr_58.5_Cu_15.6_Ni_12.8_Al_10.3_Nb_2.8_) represents a refined version of the earlier composition (Vit106), with slight adjustments to the Zr–Nb and Cu–Ni–Al ratios and demonstrates exceptional GFA and remarkable thermal stability against crystallization^9^. Consequently, Vit106a stands out as the most promising candidate among Be-free Vit alloys for forming an amorphous phase at lower cooling rates. However, it has so far only been successfully produced in relatively small casting thicknesses, specifically, spheres with diameters of 1.5 mm^9^, and 2–3 mm^10,11^. Therefore, further optimization is required to enable large-scale manufacturing and the production of components with greater casting thicknesses.

Accurate knowledge of the thermophysical properties of liquid alloys is increasingly essential for manufacturing of BMGs, as numerical simulations of casting processes are key to process optimization. The simulation requires several input parameters, including constant material properties such as the glass transition temperature (*T*_*g*_), liquidus temperature (*T*_*L*_), and latent heat of fusion (*ΔH*_*f*_), as well as temperature-dependent thermophysical properties. These include thermal conductivity (*λ*), density (*ρ*), surface tension (*σ*), viscosity (*η*), total hemispherical emissivity (*ε*), and specific heat capacity (*c*_*p*_)^12–15^. However, investigating the thermophysical properties of liquid BMGs at elevated temperatures is particularly challenging. Most BMGs are based on alloy systems that are highly reactive in their molten state and prone to cascading crystallization events, either due to impurity inclusions or interactions with container walls. To mitigate these issues, levitation techniques are commonly used in ground-based experiments, allowing for containerless processing using electromagnetic levitation (EML) or electrostatic levitation (ESL)^16–22^. In ground-based EML experiments, the effects of gravity must be counteracted by electromagnetic forces, simultaneously coupling levitation and heating, which makes significant undercooling of liquids difficult. The strong electromagnetic forces required for levitation induce stirring within the melt, which hinders fundamental measurements such as diffusion. Additionally, containerless melting on Earth often suffers from convective flows caused by temperature gradients within the liquid. These flows, driven by gravity, levitation forces and Marangoni convection, can compromise measurement accuracy^23^. Fecht and Johnson introduced an alternating current (AC) pulse heating technique in an EML system, which when applied in microgravity enables more precise thermodynamic measurements of supercooled melts^23^. Consequently, under microgravity conditions, metallic samples can be levitated and melted without container contact, preventing contamination and allowing accurate thermophysical property measurements of the liquid state.

Many measurements of the thermophysical properties of BMGs in the liquid state have already been conducted aboard the ISS, leveraging the long-term microgravity conditions provided by the ISS-EML^16–20^. In this study, we measure the thermophysical properties of a Vit106a alloy (sphere of 6.5 mm diameter) in the liquid state across multiple melt-solidification cycles under microgravity conditions aboard the ISS, focusing on surface tension (*σ*), viscosity (*η*), total hemispherical emissivity (*ε*) and specific heat capacity (*c*_*p*_). These results are compared with ground-based measurements of the same alloy. After returning to Earth, we analyze the solidified structure and morphology using X-ray diffraction (XRD) and scanning electron microscopy (SEM). While the current study focuses on providing thermophysical data of one Vit106a sample, more work remains to model and control the processing conditions for large-volume production.

## Methods

### Sample preparation

Three Vit106a alloy samples (Flight sample named FS and two spare one, respectively, Sp-1 and Sp-2) were prepared using a two-step procedure^24^, where a Ni-Nb prealloy (Prealloy 1) was made with a composition Ni-74.27 wt% and Nb-25.76 wt%, which was then cast into 5 mm diameter rods. These rods were sectioned into precise weight slices for the final alloying step. For casting of the Vit106a alloy samples, ingot of 2.2 gr weight was produced by alloying Prealloy 1 with the elements Zr, Cu and Al. The alloy ingot was remolten several times to achieve homogenous composition. The samples were cast from the ingots into 6.50 ± 0.20 mm spheres in a water-cooled copper mold. Runners and feeders were cut off and sent to an external laboratory for oxygen analysis via the LECO hot gas extraction method. For all flight samples, i.e., the flight sample itself and the two spares, the oxygen concentration was obtained as ≤0.001 mass% (10 ppm), which is considered excellent for a Zr-based alloy.

The actual surface composition was further analyzed by energy-dispersive X-ray spectroscopy (EDX) using a scanning electron microscope on two samples FS and Sp-1. The composition closely matched with the nominal composition of Zr_58.5_ Cu_15.6_Ni_12.8_Al_10.3_Nb_2.8_, with observed maximum deviations for the two measured samples of approximately +1.7 at% for Zr, −0.1 at% for Ni, −0.9 at% for Al, −0.5 at% for Cu, and +0.6 at% for Nb (see Supplementary Fig. 1 for atomic composition data). Only the FS sample was analyzed in this study. The obtained metallic sphere measured 6.51 mm in diameter with a density of 6.73 g.cm^-^³.

### Oscillation drop method

The oscillating drop technique, often combined with levitation, is widely used to measure the surface tension and viscosity of liquids. This method is based on Rayleigh’s theory, which describes surface tension-driven oscillations of a spherical, force-free liquid droplet^25^. In this experiment, the sample is heated, melted, and overheated, then allowed to cool. During cooling, a short heater pulse induces surface oscillations, which are recorded by a high-speed video camera. Using dedicated software, the average sample diameter (a) and the time-dependent deviation (δ(t)) from the equilibrium diameter are determined. The time-dependent radius $R(t,\vartheta ,\varphi )$ of a liquid droplet can be expressed as:

1 $$R\left(t,\vartheta ,\varphi \right)=\mathop{\sum }\limits_{l}\mathop{\sum }\limits_{m=-l}^{+l}{a}_{l,m}\left(t\right){Y}_{l,m}(\vartheta ,\varphi )$$

with the coefficients ${a}_{l,m}\left(t\right)$ and the spherical harmonics ${Y}_{l,m}(\vartheta ,\varphi )$ of order l with modes *m* =-l, …+l. For the *Y*_*2,m*_ harmonic, the *m* =+/−2, +/−1, 0 modes are degenerate in the force free case, as it can be achieved in micro gravity. The surface oscillation frequency ${\omega }_{0}$ for the *Y*_*2,m*_ modes is related to the surface tension by Rayleigh’s equation^25^. Due to the symmetry of the excitation, the *Y*_*2,0*_ mode is the mode which is predominantly excited when short heater pulses are used to excite the droplets surface oscillations. An aspherical sample shape, as well as sample rotations can remove the degeneracy of the oscillation modes. Under microgravity conditions aboard the ISS, the sample exhibits surface oscillations at a single frequency (see Supplementary Fig. 2). Since the oscillation amplitudes were small enough, the surface tension is related to^25^

2 $$\sigma \left(T\right)=\frac{3}{8}\pi {f}_{{osc}}^{2}M$$

where *M* is the sample mass, and *f*_osc_ the observed oscillation frequency. The recorded oscillation amplitudes are analyzed using a Fourier transformation algorithm to determine the oscillation frequency (*f*_osc_) as a function of temperature. Internal friction of the liquid during oscillation leads to considerable dampening of the oscillations. As a consequence, an exponential decay of the oscillations are observed, with the amplitude *A*(*t*) following the form *A*(*t*)=*A*_0_ exp(-*t* / *τ*). The damping time constant *τ* and the viscosity *ƞ* are related by^26^

3 $${\rm{\eta }}=\frac{3}{20\pi }\frac{M}{R}\frac{1}{\tau }$$

where *R* is the radius of the sample. Further details of the methodology can be found in ref. ^16^.

### AC modulation calorimetry

The measurement of specific heat, and total hemispherical emissivity was conducted following modulation calorimetry, adopted for electromagnetic levitation, as introduced by Fecht et al.^27^ and further developed by Wunderlich et al.^28^. Details of the method can also be found in ref. ^16^. The power, dissipated in the sample in order to heat it, is modulated in the form:

4 $${P}_{H}\left(t\right)={P}_{H0}+{\Delta P}_{{av}}+{\Delta P}_{mod}\sin ({\omega }_{mod}t+{\varphi }_{0})$$

Where ${P}_{H0}$ is a constant heating power, ${\Delta P}_{{av}}$ is denoting a stepwise increase in constant heating power, ${\Delta P}_{mod}$ is the amplitude of power modulation at the modulation frequency ${\omega }_{mod}.$ The temperature response for such a power modulation is given as:

5 $$T\left(t\right)={T}_{0}+\Delta {T}_{{av}}\left[1-\exp \left(-\frac{t}{{\tau }_{1}}\right)\right]+\Delta {T}_{mod}\sin ({\omega }_{mod}t+{\varphi }_{1})$$

Where ${T}_{0}$ is the equilibrium temperature reached for the heating power ${P}_{H0}$, ${\Delta T}_{{av}}$ is the equilibrium temperature reached after a step increase of heating power by ${\Delta P}_{{av}}$ and ${\Delta T}_{mod}$ is the amplitude of temperature modulation. The term $\Delta {T}_{{av}}\left[1-\exp \left(-\frac{t}{{\tau }_{1}}\right)\right]$ is the response to a stepwise increase of the average heating power ${P}_{H0}$ by ${\Delta P}_{{av}}$. The internal heat distribution is related to a time constant ${\tau }_{2}$ which gives rise to a phase shift $\varphi ={\varphi }_{0}-{\varphi }_{1}$ between the temperature and power. The samples heat capacity can be obtained by:

6 $${C}_{p}=\frac{{\Delta P}_{mod}}{{\omega }_{mod}\Delta {T}_{mod}}f({\omega }_{mod},{\tau }_{1},{\tau }_{2})$$

where $f({\omega }_{mod},{\tau }_{1},{\tau }_{2})$ is a correction function, considering the external and internal relaxation constants ${\tau }_{1}$ and ${\tau }_{2}$. It should be noted that the value of *C*_*p*_ does not directly depend on the absolute value of *T*, nor on the total hemispherical emissivity $\varepsilon$ but rather on the amplitude of the temperature modulation ${\Delta T}_{mod}$. The external relaxation time ${\tau }_{1}$ is related to the total hemispherical emissivity as

7 $${\tau }_{1}=\frac{{C}_{p}}{16\,\pi \,{R}^{2}\varepsilon {\sigma }_{B}{T}^{3}}$$

Where *R* is the sample radius, ${\sigma }_{B}$ the Stefan-Boltzmann constant. As shown in Eq. (7), the determined total hemispherical emissivity *ε* is highly sensitive to errors in the temperature T. The used pyrometer to obtain the temperature is sensitive in the wavelength range 1.45–1.80 µm and offers a resolution of better than 0.1 K. Prior to the space experiments, the spectral emissivity was determined in a ground-based experiment and was around 0.27. The measured temperature T on the ISS was further corrected by comparing the liquidus temperature obtained in space with its previously determined ground-based value. When necessary, the measured temperature was adjusted according to the following relation:

8 $$\frac{1}{{Tcorrect}}=\frac{1}{{Tmeasured}}+\frac{1}{{Tcorrect\; liquidus}}-\frac{1}{{Tmeasured\; liquidus}}$$

Where *T*_*correct*_ denotes the corrected (real) temperature, T_*correct liquidus*_ refers to the liquidus temperature determined from ground-based experiments and reported in the literature, and *T*_*measured liquidus*_ corresponds to the liquidus temperature measured on the ISS from the isothermal plateau observed in the temperature–time profile. The given error bars in $\varepsilon$ are a result of the error propagation of the uncertainty of the *C*_*p*_, as well as the uncertainty in temperature.

### X-ray diffraction (XRD)

XRD measurements were conducted in Bragg-Brentano geometry (θ–2θ) using an Empyrean diffractometer (Malvern Panalytical) equipped with a PIXcel3D-Medipix3 detector operating in line scan mode. The system was set to 45 kV and 40 mA, utilizing Cu-Kα radiation (combined Kα₁ and Kα₂). The divergence slit and the beam were selected to obtain a 20 mm^2^ beam size on the sample. The integrated area of the XRD pattern were normalized to account for the effective beam coverage during mapping. The mapping was performed relative to the center of the sample, with XRD scans acquired at X = ± 2 mm and Y = ± 2 mm.

### SEM imaging

The microstructural analysis of the samples was performed using a Zeiss Gemini SEM, equipped with a field emission gun (FEG) for high-resolution imaging. Secondary electron (SE) and backscattered electron (BSE) imaging modes were utilized to examine surface morphology and compositional contrast, respectively. Imaging of the cross-section was conducted to assess grain structure, void distribution, and phase homogeneity.

## Results

### Surface tension and viscosity

**Fig. 1:**
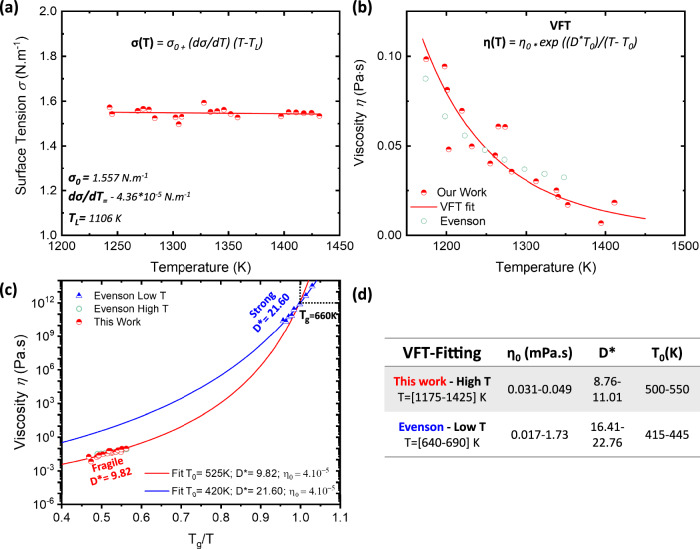
Surface tension and viscosity of liquid Vit106a. **a** Surface tension (σ) and **b** viscosity (η) of the liquid Vit 106a droplet measured abord the ISS-EML facility using the oscillating drop method. **c** Angell plot presenting comparing our viscosity (η) data with that obtained by Evenson at both low (near Tg) and high temperatures. The red and blue line correspond to VFT fits, corresponding fitting parameters displayed in the graph. **d** Fitting parameters (η_0_, D* and T_0_) obtained by varying T_g_ for the high-temperature range and η_0_ for the low-temperature data.

Understanding surface tension *σ* and viscosity *η* is crucial for controlling the solidification process of BMGs because they directly influence glass formation and casting quality. On the electromagnetic levitator on board the ISS, the viscosity *η* of the stable metallic liquid and its surface tension (*σ*_*L-G*_) are obtained using the oscillating drop method. The surface tension *σ*_*L-G*_ influences the shape, stability, and wetting behavior of the molten alloy. The surface tension was measured in vacuum and derived from the samples surface oscillation frequency following an excitation pulse. The surface tension for the temperature range [1240–1430] K is presented in Fig. 1a and is nearly constant over the studied temperature interval. A linear fit of the data results in the following temperature dependence of the surface tension:

9 $${\sigma }_{L-G}(T)={\sigma }_{0}-{\rm{d}}\sigma (T)/{\rm{d}}{T}^{* }(T-{T}_{L})$$

Where *σ*_*0*_ = 1.557 ± 0.018, d*σ*(*T*)/d*T* = −(4.36 ± 7.65) × 10^–5^ and *T*_*L*_ = 1106 K. The surface tension data reported in Fig. 1 shows that the surface tension of the Vit106a is relatively like other Zr-based metallic glass formers such as Vit105 (Zr_52.5_Ti_5_Al_10_Ni_14_Cu_17.9_) or a Zr_50_Cu_50_ alloy measured in the same conditions^6,20^.

The viscosity *η* of molten Vit106a, measured aboard the ISS within the temperature range [1175–1425] K, is shown in Fig. 1b. The data from Evenson et al. obtained on a Vit106a alloy using Couette concentric cylinder viscometer near the equilibrium melting temperature is also added^29^. Our data are also presented in an Angell plot in Fig. 1c, together with Evenson’s high- and low-temperature data^29,30^. The low-temperature data were obtained near *T*_*g*_ and correspond to equilibrium viscosity measurements determined using three-point beam bending. The temperature-dependent viscosity *η*(T) of liquid can be described by the empirical Vogel–Fulcher–Tammann (VFT) equation^31^:

10 $$\eta \left(T\right)={\eta }_{0}\exp \left(\frac{D* {T}_{0}}{T-{T}_{0}}\right)$$

where *η₀* represents the viscosity in the high-temperature limit, typically in the liquid state well above the melting temperature. *T₀* is an extrapolated temperature located below *T*_*g*_ at which the viscosity would theoretically diverge to infinity. D* is the kinetic fragility parameter, which quantifies the deviation from Arrhenius behavior and determines whether the liquid exhibits strong or fragile glass-forming characteristics.

The VFT fit for our data is represented by a continuous red line, obtained using a *T*_*g*_ value of 660 K for Vit106a, ensuring that the fit yields a viscosity of 10^12^ Pa.s at *T* = *T*_*g*_. This results in the parameters D* = 9.82, *T*_*0*_ = 525 K, and *η₀* = 4.10^−5 ^Pa.s. The obtained *η₀* value aligns with expectations based on the relation *η₀* = *Na.h/ V*_*m*_, where *N*_*a*_ is Avogadro’s number, *h* is Planck’s constant, and *V*_*m*_ is the molar volume of the alloy^32^. The D* value determined here is consistent with the previously reported D* = 10 for Vit106a in ref. ^29^. Besides, the *T*_*0*_ value obtained is similar to the one obtained for a Vit106 (*T*_*0*_ = 524.7 K*)* and Vit105 *(T*_*0*_ = 521 K*)*^32^. A VFT fit for liquid Vit106a reported in ref. ^21^ using ESL experiments over the temperature range T = 1550 K to 1100 K yielded a D* = 12.98 (5.60 in base Log), *T*_*0*_ = 575 K, and *η₀* ≈ 3.31 × 10^−4 ^Pa·s. These parameters differ from ours, however their values remain in the same order of magnitude. The VFT fit applied to Evenson’s low-temperature data, with *η₀* fixed at 4.10^−5 ^Pa.s, yields D* = 21.60 and *T₀* = 420 K, which is consistent with the values reported in ref. ^29^. However, if *T*_*0*_ is fixed at 436.8 K, as given in ref. ^33^, the fitting parameters change to D* = 17.96 and *η₀* = 5.10^−4 ^Pa.s. This value is close to the D* = 19.7 obtained from the VFT fit of the enthalpy relaxation time using *T*_*0*_ = 436.8 K.

It is important to note that in VFT fitting, the D* parameters are strongly influenced by the choice of *T*_*g*_ in the high-temperature range, while the evaluated *T*_*g*_ can vary across different studies and as a function of the cooling rate^34^. Conversely, in the low-temperature range, the fit is more sensitive to *η₀*, which should typically be close to 10^−4 ^Pa.s for all materials^35,36^. This sensitivity arises because high-temperature data are measured far from *T*_*g*_, and low-temperature data are far from *η₀*, meaning that even slight variations in these values can lead to significant deviations in the fit. Therefore, the range of η₀, D* and *T₀*, values obtained for the fitting parameters is provided in the table shown in Fig. 1d. This range considers our data with *T*_*g*_ values between 650 K and 670 K^30,33,34^, and for the low-temperature range, *η₀* is maintained between 10^–3^ and 10^−5 ^Pa.s (see Supplementary Fig. 3). Taking into account variations in prefixed parameters such as *T*_*g*_ and *η*_*0*_, the resulting kinetic fragility parameter D* from the VFT fit can vary by ~25–40% within the same temperature range. However, the D* values obtained from the high- and low-temperature ranges differ significantly and do not overlap. This indicates that the Vit106a liquid possesses different kinetic fragility D* in these two respective regimes. This difference in kinetic fragility D* between the high- and low-temperature has been reported in several studies and is linked to the strong-to-fragile transition observed in Zr-based glass-former alloys.

### Total hemispherical emissivity and specific heat capacity

In the EML experiment, the *ε* and *c*_*p*_ value is obtained from AC modulation calorimetry experiments and from the increase in the average temperature upon application of heating power modulation (see section experimental for more details on the method). The total hemispherical emissivity *ε* of the liquid melts is a property important for the description of the radiative cooling of a liquid. The specific heat capacity *c*_*p*_ of BMG-forming alloys in the liquid state also plays a crucial role in controlling the cooling rate necessary for proper solidification. Additionally, it serves as an essential parameter in conjunction with other thermodynamic properties to determine Δ*G*, the Gibbs free energy difference between the liquid and crystalline phases, which is fundamental for nucleation modeling. The advantage of modulation calorimetry calibration as compared to pulse heating experiments is that no knowledge of *ε* is required.

**Fig. 2:**
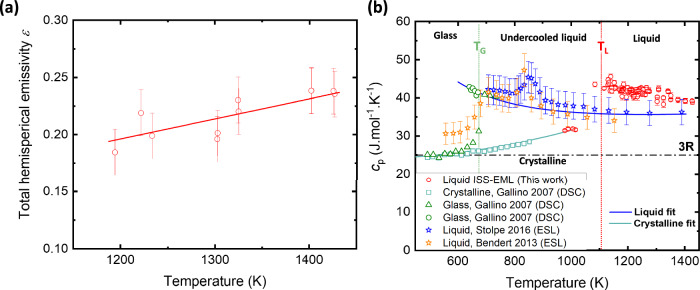
Emissivity and heat capacity of liquid Vit106a. **a** Total hemispherical emissivity (ε) and **b** specific heat capacity (c_p_) of the liquid Vit 106a droplet measured abord the ISS-EML facility using AC modulation calorimetry. For comparison, the c_p_ data obtained via differential scanning calorimetry (DSC) at low temperatures from Gallino’s work, as well as the c_p_ values derived from temperature-time profiles using the c_p_ /ε ratio (ε = 0.22 ± 0.02) in the studies by Bendert and Stolpe, are also included.

Figure 2a shows the total hemispherical emissivity *ε* value obtained for the Vit106a sample measured between T = 1430 K and T = 1120 K. The *ε* value vary from *ε* = 0.18 ± 0.02 to *ε* = 0.24 ± 0.02. Figure 2b presents the *c*_*p*_ value obtained by AC modulation calorimetry, along with the *c*_*p*_ value obtained by calorimetry in the work of Gallino et al.^33^. The *c*_*p*_*/ε* value obtained using the Stefan-Boltzmann law from the temperature-time profile of electrostatic levitated (ESL) Vit106a droplets, measured in the work of Stolpe et al. and Bendert et al. on ground-based experiment, are also added in the *c*_*p*_ graph by considering an average value of *ε* = 0.22 ± 0.02^10,11^. No new terrestrial data were collected in this study, ground-based results were used solely for comparison purposes.

Our data obtained above *T*_*L*_ show an increase of *c*_*p*_ from 38 to 42 J.mol^−1^. K^−1^ as the temperature decreases from 1430 K to 1115 K. These values measured in the stable liquid state are higher than the one reported by Stolpe et al. around 36 ± 4 J.mol^−1^. K^−1^ and the extended fit of the low-temperature data for the undercooled liquid measured by Gallino et al. The three data points obtained between 1020 K and 980 K are measured within the crystalline state and match quite well with the extended fit of the low-temperature data for the crystalline state measured by Gallino et al. Measurement of the *c*_*p*_ in the undercooled liquid state was not possible, as our Vit 106a sample exhibited a strong tendency to crystallize before reaching this state, preventing data acquisition.

The appearance of peaklike anomalies in *c*_*p*_*/ε* around 800–900 K is due to enthalpy release in the liquid state (also observed in a Vit106 alloy in ref. ^11^) and has been attributed to a partial crystallization of the alloy in the work of Bendert et al. Conversely, the same peak was attributed to a transition from a less ordered high temperature (HTL) to a more ordered low temperature liquid phase (LTL) in the work of Stolpe et al. and has been correlated to the strong-to-fragile transition observed in viscosity measurement.

### Solidification behavior

**Fig. 3:**
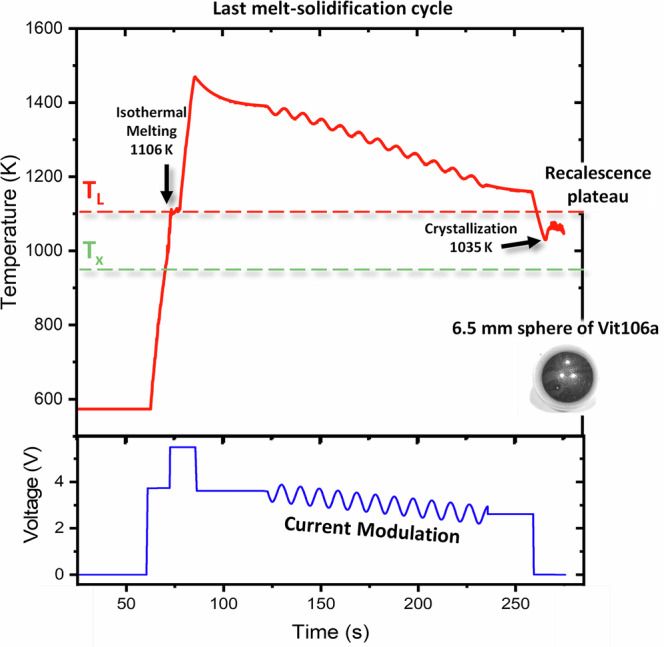
Temperature-time profile of the liquid Vit106a droplet recorded by a pyrometer during the final melt-solidification cycle aboard the ISS-EML before returning to Earth. The applied heater voltage is shown in blue. The experiment was conducted in a high-purity (99.9999%) helium atmosphere. The recalescence plateau marks the onset of crystallization at 1035 K, whereas literature reports suggest an expected crystallization temperature of around 950 K.

Figure 3 shows the temperature-time profile of the last melt-solidification cycle conducted aboard the ISS. During this last cycle, the heating rate of the sample is 48 K.s^−1^ and the alloy melts at 1106 K, where a temperature plateau is observed. This plateau occurs because the energy supplied to the system is entirely used to fully melt the sample, preventing further temperature increase during this phase transition. The value of melting temperature is the same as the one found in the literature of 1103 K in ref. ^9^ and 1109 K in ref. ^33^. The sample is overheated above the melting temperature up to 1470 K and the heater current modulation was performed within the temperature range of 1400 K to 1150 K. The molten alloy is then cooled from 1150 K by switching the heater voltage to 0 V, allowing for free radiative cooling at an average rate of 18 K.s^−1^. The thermal profile reveals the formation of a recalescence plateau, which marks the onset of crystallization at 1035 K. The recalescence plateau is driven by the exothermic heat release (latent heat of fusion) associated with the transformation from the undercooled liquid state to the crystalline phase. The crystallization temperature of the undercooled liquid Vit106a is significantly higher than the one reported in the work of Hays et al. (~950 K)^9^. Furthermore, in the study by Hays et al., the critical quenching rate required to form an amorphous phase was estimated at 1.75 K.s⁻¹, with the “nose” of the TTT diagram located at 900 K. In our case, the quenching rate near the crystallization temperature is 16 K.s⁻¹, approximately nine times higher than the critical rate reported by Hays et al. This should, in principle, have prevented crystallization. This issue will be discussed in more detail later.

**Fig. 4:**
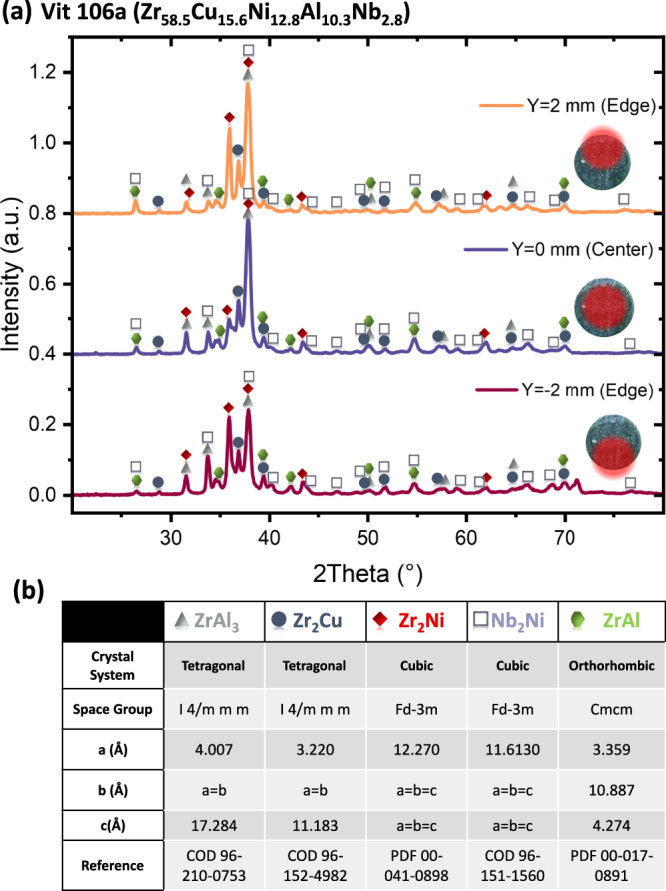
XRD analysis after solidification of the Vit106a sample. **a** XRD patterns obtained from scans across the cross-section of the sample produced aboard the ISS. Small schematics accompanying the graphs illustrate the X-ray beam position on the sample for each corresponding pattern. **b** Table summarizing the most probable crystalline phases identified in the Vit106a alloy.

After the thermophysical investigation conducted abord the ISS, the Vit106a sample was returned to earth. A 200 μm cross-section taken at the middle of the 6.5 mm diameter BMG spheres was characterized by XRD to investigate its structure. Figure 4a presents the XRD patterns obtained at different beam positions across the sample. The XRD patterns exhibit a high number of sharp, high-intensity diffraction peaks in the 2*θ* range of 30° to 45°, and low-intensity diffraction peaks at higher angles, with no detectable amorphous contribution which confirms that the sample is fully crystalline. This result indicates that, despite a relatively high cooling rate of 16 K.s⁻¹, the quenching conditions were still insufficient to suppress crystallization. In fact, if the sample were approaching the critical cooling rate, we would expect at least a partially amorphous structure. The complete absence of an amorphous signal suggests that the sample remained far from the critical quenching threshold required for glass formation under these conditions.

Phase identification was realized using the HighScore software using the COD (Crystallography Open Database) and PDF (Powder Diffraction File from the International Centre for Diffraction Data) databases and reveals the presence of various binary intermetallic compounds within the Zr-Al, Zr-Cu, Zr-Ni and Nb-Ni systems. The most probable crystalline phases are identified with different symbols in the XRD patterns and are summarized in the table in Fig. 4. These phases include tetragonal ZrAl₃ and orthorhombic ZrAl. However, the Zr-Al system contains numerous compounds that could match multiple diffraction peaks, such as tetragonal Zr₃Al₂, orthorhombic Zr₂Al₃, Zr₅Al₃, and hexagonal Zr₄Al₃ and Zr₃Al₂. Therefore, the presence of multiple crystalline phases within the sample is likely, given the wide range of possible compositions in the Zr-Al binary system. In contrast, the Zr-Cu system features only the tetragonal Zr₂Cu phase, while the Zr-Ni and Nb-Ni systems contain exclusively the cubic Zr_2_Ni and Nb₂Ni phases.

While the diffraction peaks relative to these crystalline phases are always present at different position throughout the sample, their relative intensity is not homogeneous and varies significantly depending on the scanned region. However, due to the overlap of diffraction peak positions among these phases, precise phase quantification remains challenging, if not impossible. All the phases identified via XRD appear to be binary compounds, with no ternary phases detected. Similar crystalline phases, such as Zr₂Cu, ZrNi₂, Zr_3_Al_2_ and Zr_4_Al_3_ have been observed in crystalline Vit106 alloys^37^. However, despite the close atomic composition of this alloy to Vit106, it does not exhibit exactly the same phases after solidification, for instance any match is possible for the ZrCu phase^37^.

**Fig. 5:**
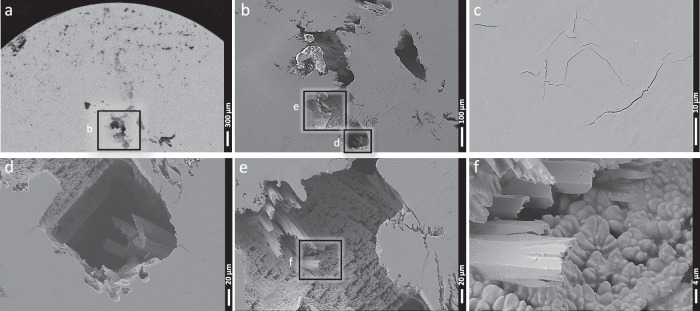
SEM secondary electron (SE) images of the cross-section taken at the center of the Vit106a sphere, captured at various magnifications. **a** Low-magnification overview of the entire cross-section (×62). **b** Zoomed view of the central region from (**a**) (×241). **c** Localized cracks observed in specific regions of the cross-section (×3.39K). **d** Higher magnification of the region highlighted in (**b**) (×1.05K). **e** Further magnification of a different area within (**b**) (×901). **f** Detailed zoom of the region shown in (**e**) (×4.36K). The images reveal pronounced voids near the center of the sphere and expose the internal microstructure, characterized by two distinct types of crystallites.

Figure 5 presents SEM images of the cross-section taken from the center of the sphere. Large, deep voids were primarily observed in the core region, along with some cracks. These features are likely associated with crystallization shrinkage. This feature suggests that crystallization started at the edges of the sample rather than homogenously in the bulk, leading to insufficient material remaining at the center resulting in the formation of deep and huge voids. Although SEM data from a terrestrially processed reference sample subjected to identical thermal conditions is unavailable, ground-based Vit106a samples are typically fully amorphous prior to processing and do not exhibit such voids. A comparative study of glass samples processed on Earth and in space reported similar morphology and short-range order in the glass^38^. Moreover, comparable void structures have been observed in other fully crystalline materials processed in microgravity, such as Ti64^19^. These observations suggest a possible link between void formation and crystallization, although the phenomenon may also arise as an artifact of microgravity. Indeed, the absence of buoyancy-driven convection in microgravity may subtly influence heat and mass redistribution during solidification, potentially affecting void morphology.

A magnified view of these voids, shown in the rightmost images, reveals the alloy’s microstructure after solidification. The microstructure consists of two main types of crystallites. Large, elongated crystallites, exhibiting a rectangular morphology with widths ranging from 5 to 15 μm and visible lengths of up to 80 μm. These structural features suggest they could correspond to the tetragonal Zr_2_Cu and ZrAl₃ or other tetragonal/orthorhombic Zr-Al phases. These crystallites contribute to a significant portion of the microstructure. Smaller, more uniform crystallites are also observed. These structures have a symmetric, cubic-like shape with sizes around 4 μm and are likely associated with the cubic Zr_2_Ni and Nb_2_Ni phases. No significant contrast variation was observed (see Supplementary Fig. 4) using the Electron Backscattered Detector (EBSD), which is highly sensitive to the atomic number (Z) of elements. This is likely since all identified phases have comparable Z values, as they primarily consist of Zr atoms.

## Discussion

In Zr-based metallic glasses, surface tension generally decreases as temperature increases^6,20,39^. In our study, the surface tension of the liquid Vit106a, as shown in Fig. 1a, remains nearly constant with increasing temperature. However, the studied temperature range is too narrow to fully characterize the overall trend. Surface tension gradients arising from temperature differences within a liquid drive fluid motion from hotter regions (with lower surface tension) toward cooler regions (with higher surface tension), a phenomenon known as Marangoni flow. Therefore, the observed weak *σ(T)* variation for Vit106a implies a correspondingly weak Marangoni effect. This weak Marangoni flow may be beneficial for BMG-forming ability, as it reduces the emergence of dynamic heterogeneities within the liquid that can promote crystallization during cooling. The surface tension of Vit106a is nearly identical to that of Vit105 (Zr_52.5_Ti_5_Al_10_Ni_14_Cu_17.9_) and Zr₅₀Cu₅₀ alloys. The surface tension values obtained for Zr-based alloys differ significantly from those of Fe-based and Pd-based BMG measured in the same conditions, highlighting the strong influence of chemical composition on surface tension^17,18^. In the binary Zr–Cu system, for example, surface tension increases markedly with higher Zr content (and correspondingly lower Cu content). Similar trends have been observed in the Cu–Fe–Si and Zr–Ti–Cu ternary systems, where significant variations in surface tension occur with changes in the molar fractions of the constituent elements^40,41^. The similarity in *σ* between Vit106a and Vit105 is expected, as they share close Zr/Cu ratio (Zr/Cu = 3.75 for Vit106a, and 2.93 for Vit105). In contrast, Zr₅₀Cu₅₀ has a much lower Zr/Cu ratio (Zr/Cu = 1) compared to Vit106a and Vit105, yet it exhibits a similar *σ* value. However, all the three alloys have a similar overall zirconium content around 50–60 at.%. This suggests that the relative proportion of Zr plays the most significant role in determining *σ* in Zr-based BMGs.

Bulk metallic glass-forming liquids are densely packed, resulting in high viscosity and sluggish crystallization kinetics compared to other metallic liquids. In the VFT equation, the kinetic fragility parameter D* quantifies how strongly the temperature dependence of viscosity or relaxation time deviates from Arrhenius behavior. Lower D* values correspond to more fragile liquids, which exhibit a more rapid increase in viscosity as the temperature approaches the glass transition. In this study, Vit106a exhibits a kinetic fragility parameter of D* = 9.8 in the high-temperature range of 1200–1400 K, similar to the value measured for other Vit alloys^29,32^. A D* value of 9.8 corresponds to a more fragile-like liquid behavior, comparable to that of the fragile glass-former o-terphenyl (D* = 2), while being significantly lower than that of the strong network glass-former SiO_2_ (D* > 100). However, when measured near the glass transition temperature *T*_*g*_, the fragility of Vit106a increases significantly, with a value of D* = 21.6. This substantial difference in D* between the high- and low-temperature range arises because most Zr-based metallic glass-forming liquids exhibit a fragile dynamic response above the melting point, typically with D* decreasing to values around 10. However, upon undercooling the liquid closer to their *T*_*g*_, their effective fragility significantly increases.

In Vit 1 the underlying mechanism of the fragile-to-strong transition has been attributed to a liquid-liquid structural transition (LLT) that has been observed through a combination of in-situ XRD and ESL^42^. This LLT is attributed to changes in short-range order (SRO) and medium-range order (MRO) within the liquid, which significantly influences its viscosity. Additionally, the viscosity of liquid Vit 1 exhibits pronounced shear-thinning behavior, which has also been ascribed to the disruption of SRO/MRO structures under the influence of shear and elevated temperatures^43^. In Vit 1, the LLT exhibits a pronounced thermal hysteresis, occurring between ~1100–1200 K upon heating and 760–830 K upon cooling. This hysteresis is reflected in the viscosity behavior of the liquid^42,43^.

LLTs are often associated with exothermic peaks observed upon cooling in *c*_*p*_*(T)* measurements. For Vit106a, an exothermic peak is observed around 800–900 K in the *c*_*p*_*(T)* curve (as shown in Fig. 2b), though its origin remains a subject of debate. Bendert et al. interpreted it as evidence of partial crystallization, estimating a crystallized volume fraction of 10–15%^11^. In the same study, a comparable exothermic peak and accompanying change in specific volume were reported for Vit106 upon cooling, again interpreted as partial crystallization with an estimated volume fraction of 3–5%. In contrast, Stolpe et al. attributed the exothermic *c*_*p*_*(T)* peak of undercooled Vit106a to an LLT, due to the absence of crystallite signals in 2D X-ray detector images and the consistency of the measured relative heat release with previous LLTs reported in other BMG^10^. Supporting this interpretation, Li et al. associated the change in specific volume observed upon heating in liquid Vit 106 also with an LLT^44^. Consequently, the change in viscosity behavior with temperature reflected by the variation in the fragility parameter D* observed in Zr-based BMG formers is usually attributed to an LLT. This LLT is driven by the formation or destruction of SRO/MRO within the liquid, which significantly influences its viscosity.

Therefore, our study supports the existence of a fragile-to-strong transition in Vit106a alloy. However, we were unable to confirm the presence of an exothermic peak in the *c*_*p*_*(T)* curve during undercooling, as the Vit106a liquid crystallizes near its *T*_*L*_, preventing observation of this feature. The last melting-solidification cycle shown in Fig. 3 presents a recalescence plateau, which marks the onset of the crystallization process, occurring at 1035 K. This is significantly higher (Δ= +85 K) than the highest crystallization temperature reported in the TTT diagram in the work of Hays et al.^9^. Indeed, even for very slow cooling rates the TTT diagram suggests a crystallization temperature around 950 K. Besides, none of the thermal cycles performed on the Vit106a sample aboard the ISS resulted in the formation of an amorphous phase. This is unexpected, given that Vit106a has previously been reported as a highly stable glass-former, capable of vitrifying at relatively low quenching rates, as low as 1.75 K.s⁻¹. These results raise important questions about the true robustness of Vit106a’s glass-forming ability, particularly whether this ability diminishes significantly with increasing sample volume.

The lowest reported cooling rate for successful vitrification of Vit106a in ESL experiment is 1.75 K.s⁻¹, achieved for a sphere with a diameter of 1.5 mm^9^. In the study by Bendert et al., partial crystallization was suggested for a 2–3 mm diameter Vit106a sphere cooled at an estimated rate of 3–4 K.s⁻¹, although direct evidence of crystallization was not observed^11^. Stolpe et al. successfully vitrified a 2–3 mm diameter sphere at an average cooling rate of 8 K.s⁻¹ (estimated between 1100 K and 900 K) when cooled from an initial temperature of 1500 K^10^. However, the same alloy crystallized at 980 K when cooled from 1380 K, despite a slightly higher average cooling rate of 10 K.s⁻¹ (between 1100 K and 1000 K). Evenson et al. also reported that their Vit106a alloys show a recalescence at 1075 K, when cooled in a graphite crucible at a rate of 3 K.s^-1^^29^. They suggest that Vit106a alloy may be highly sensitive to heterogenous nucleation, which is not the case for Vit1 which shows no such recalescence.

It has been demonstrated that the solidification behavior of Vit alloys is very sensitive to their thermal history during processing. A study on Vit1, Vit105, and Vit106 alloys, which are closely related to Vit106a composition, has shown that the overheating temperature, the temperature above *T*_*L*_ from which quenching begins, has a significant impact on both *T*_*x*_ and the nucleation time. For instance, in Vit105, if the overheating temperature exceeds 1250 K, *T*_*x*_ is around 850 K. In contrast, if the overheating temperature remains below this threshold, *T*_*x*_ occurs between 1030 K and 1060 K^45^. Additionally, Bendert et al. also reported a similar behavior for the Vit106 and Vit106a alloys. In the initial thermal cycle, the alloys always crystallize. As the overheating temperature progressively increased, *T*_*x*_ shifted to lower temperatures and diminished with each cycle. Finally, they observed that no recalescence occurred when the sample was heated above a threshold temperature of ~1400 K^11^. Besides, samples prepared from starting materials with higher oxygen content exhibit increased crystallization temperatures, and oxygen concentrations above 500–600 ppm led to complete crystallization.

It is well known that Zr-based alloys are highly sensitive to oxygen, which can significantly alter crystallization kinetics^46,47^. Lin et al. demonstrated that the effect of overheating on Vit105 becomes more pronounced as oxygen content increases. Therefore, they attributed the effect of overheating to the melting of oxide precipitates in the liquid above a threshold temperature^47^. These oxides precipitate could act as nucleation sites and hinder deep undercooling. Their results also indicated that the overheating temperature must be exceeded during each heating/cooling cycle, implying that oxide particles re-form upon cooling and continue to influence the crystallization behavior. Interestingly, Bendert et al. observed that once this overheating threshold was surpassed, subsequent thermal cycles no longer required to overcome this threshold to successfully form a glass^11^. As a result, the behavior of these materials during processing remains not fully understood, and direct evidence for the presence of precipitated oxides is still lacking. Indeed, numerous studies have reported the influence of overheating on the glass-forming ability of Vit alloys, and it remains unclear whether this effect is solely due to residual impurities or is an intrinsic characteristic of Vit alloys. This uncertainty is reinforced by the fact that these alloys exhibit LLT, which may independently affect their thermo-physical response and therefore glass-forming ability^29–32,42–44^.

In our case, pre-flight measurements indicated that the oxygen content of the Vit106a sample was extremely low (<10 ppm), and all handling prior to launch was performed in a glove box to minimize oxidation. However, Vit 106a was the first sample to be processed in the nominal EML program and the pumping and flushing procedure was not optimal, which may have introduced some oxides particles during the first heating/cooling cycles. Therefore, we cannot rule out the presence of impurities introduced during processing in the EML system aboard the ISS, as the oxygen content was not measured after the sample’s return to Earth. During the experiment, the sample was heated to 1430 K but only quenched from 1160 K, suggesting that it may not have been significantly overheated beyond *T*_*L*_. This limited overheating may explain the premature crystallization observed in our results, where *T*_*x*_ occurs at 1035 K instead of the 950 K typically reported in the literature. The low undercooling observed in our study may be attributed either to static heterogenous nucleation occurring on oxide impurities, or to dynamic heterogeneities occurring within the bulk liquid that promote crystallization^48–50^.

Indeed, while all these crystalline phases observed in XRD are present throughout the sample, as evidenced by the consistent appearance of their diffraction peaks, the relative peak intensities and area vary across different scans realized at different locations. This suggests that the spatial distribution of these phases is slightly heterogeneous in terms of quantity and probably occurred due to heterogenous nucleation. Moreover, the presence of large voids observed at the center further supports the idea that crystallization likely initiated near the sample edges rather than occurring uniformly throughout the volume. Further investigation is required to determine whether the premature crystallization observed in this study is primarily due to oxides particles and suboptimal processing conditions, such as insufficient overheating, or results from the larger sample size. Notably, our sample had a diameter of 6.5 mm, corresponding to a volume ~80 times greater than that used in the work of Hays et al. It is well established that increasing casting thickness significantly reduces glass-forming ability, primarily due to the higher likelihood of heterogeneous nucleation sites and the lower cooling rate within the bulk liquid^51^.

In conclusion, this study provides a comprehensive characterization under microgravity conditions of the surface tension, viscosity, specific heat capacity, and total hemispherical emissivity of Vit106a in its molten state, providing critical thermophysical data for simulating the casting behavior of Zr-based BMGs. The surface tension remains nearly constant across the investigated temperature range and closely matches that of Vit105 and Zr₅₀Cu₅₀, highlighting the dominant influence of zirconium content. Viscosity measurements reveal a clear fragile-to-strong transition, with high-temperature fragility (D* ≈ 9.8) increasing significantly near the glass transition temperature (D* ≈ 21.6). XRD analysis confirms full crystallization of the sample, despite being cooled at a rate of 16 K·s⁻¹, over nine times faster than the critical cooling rate of 1.75 K·s⁻¹ reported in the literature. The crystallized phases consist of binary intermetallic compounds from the Zr–Al, Zr–Cu, Zr–Ni, and Nb–Ni systems. This premature crystallization upon undercooling may be attributed to heterogeneous nucleation, potentially promoted by insufficient overheating, as observed in previous studies on Zr-based BMGs. However, it remains unclear whether this phenomenon is driven by impurities or by the significantly larger sample volume, approximately 80 times greater than in prior studies. This raises the question of whether the very low critical cooling rate reported for smaller samples still applies at larger casting thickness.

These initial thermophysical datasets can be implemented in simulation platforms such as ANSYS or ProCAST into predictive models to optimize process parameters such as cooling rates, mold design, and heat extraction. However, thermal conductivity remains a critical missing parameter for accurate process simulation. Other thermodynamic inputs required for nucleation modeling, such as latent heat of fusion and transition temperature, are already available in the literature. A key limitation of this study is that it is based on a single processed Vit106a sample, which prevents broader statistical validation. To enable large-scale manufacturing of Vit106a, further studies should investigate the influence of sample size on the critical cooling rate, as larger volumes are more susceptible to crystallization due heterogeneous nucleation and lower cooling rate. Additionally, the impact of slight compositional variations on thermophysical properties and glass-forming ability should be examined, especially given that Vit106, a compositionally similar alloy, exhibits a significantly higher critical cooling rate (10 K.s⁻¹) compared to Vit106a (1.75 K.s⁻¹). Moreover, factors such as oxygen sensitivity and thermal history must be carefully controlled to suppress heterogeneous nucleation and achieve full vitrification in bulk geometries. These considerations are essential for defining robust processing windows and developing scalable manufacturing routes for Vit106a.

## Supplementary information

Supplementary information

## Data Availability

All data generated or analyzed during this study are included in this published article and its supplementary information files.
