# Supplementary material for: Thermophysical properties and solidification behavior of liquid Vit106a in microgravity
Source: NPJ Microgravity. 2026 Feb 17;12:26. doi: 10.1038/s41526-026-00572-6 (PMC13022327; doi:10.1038/s41526-026-00572-6)
Supplement: Supplementary file 1 — Supplementary information [file 41526_2026_572_MOESM1_ESM.pdf]

Supplementary Information (SI)

Sample preparation

For the preparation of the sample production a dedicated effort was made to select low oxygen Zr from different sources and to quantify a potential contribution of the casting process to the oxygen concentration of the cast product. To that end low-oxygen zirconium (Zr) from different sources was selected and tested for potential oxygen uptake during casting. Zr pieces from one supplier were surface-polished, stored, and packed under high-purity Ar in a glove box, then arc-melted into 5 mm rods. Similarly, Zr-milled chips from another supplier were first melted into an ingot, then cast into rods without breaking the protective atmosphere. Four rods from each source were produced, and four cut samples were sent for oxygen analysis via LECO hot gas extraction.

All Zr samples showed an average oxygen concentration of 0.0047 ± 0.001 wt%. Within a confidence level of ≤ 0.001 wt%, no significant oxygen increases due to melting or casting was detected.

Sample	Zr at%	Ni at%	Al at%	Cu at%	Nb at%
**FS** (Flight Sample)	59.6	12.7	9.5	15.1	3.13
**Sp-1** (Spare Sample 1)	60.2	12.8	9.4	15.3	3.40
**Nominal**	**58.5**	**12.8**	**10.3**	**15.6**	**2.8**

Figure S1 *– Atomic composition measured with EDX on two as-cast Vit106a sphere. FS is the Vit106a sample that has been processed during this study*

Oscillation Drop method

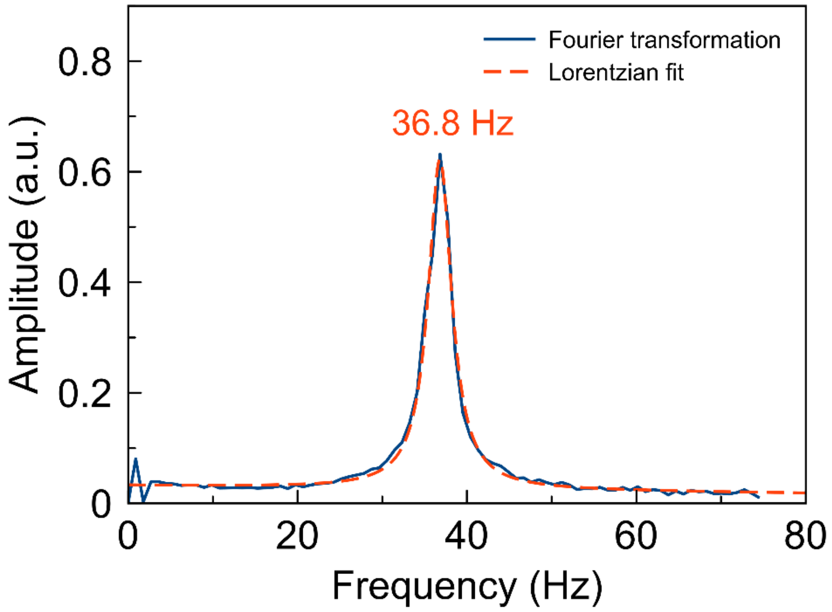

Figure S2 *– Fourier transform of a representative time dependent radius variation recorded during surface oscillations*

VFT model

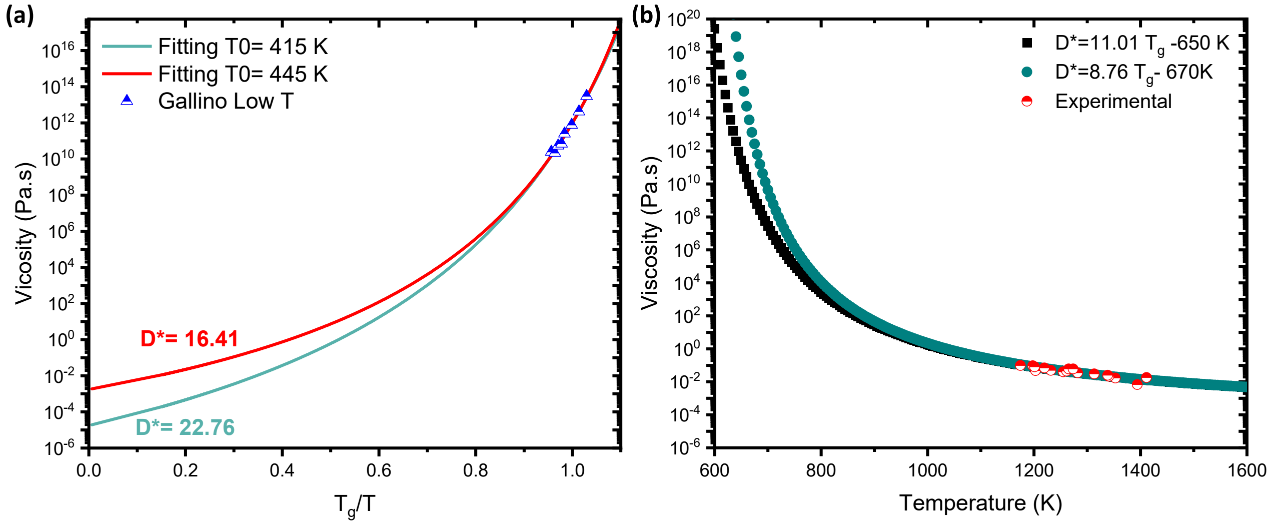

Figure S3 – (a) *Fitting of Gallino's low-temperature data yields different kinetic fragility indices (D*) depending on the chosen η_0 limit was fixed, either at 10^{-3} Pa.s or 10^{-5} Pa.s* ***(b)*** *Fitting of our data at high temperature giving different D* depending on the T_g value preselected.*

SEM images

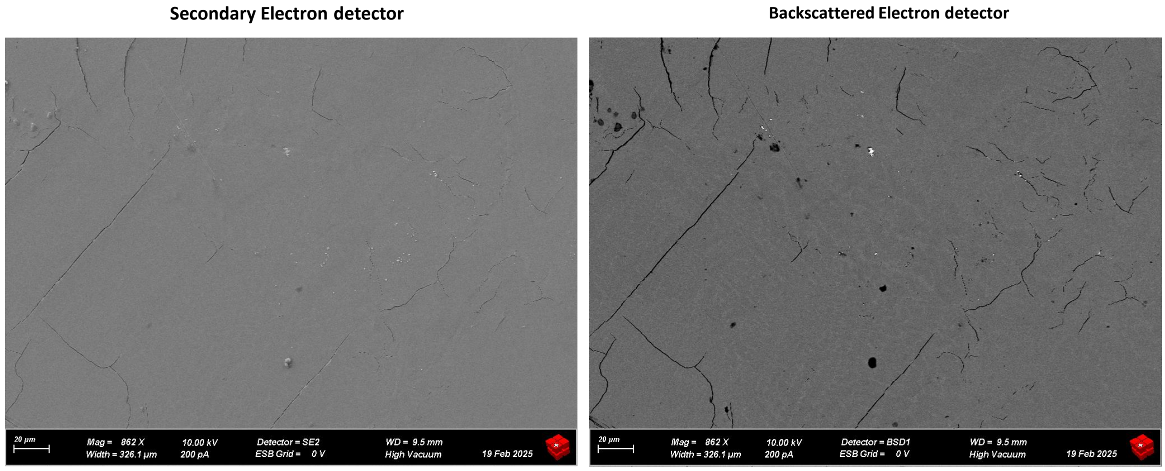

Figure S4 – *SEM image of a cross-section of the Vit106a sphere acquired using either a secondary electron detector or a backscattered electron detector.*
